# Neuroanatomical diversity in *Teleocichla* with new volumetric and histological insights into the encephalon of *Teleocichla monogramma* Kullander 1988

**DOI:** 10.1002/ar.70095

**Published:** 2025-11-13

**Authors:** Renan Leão‐Reis, Keila Xavier Magalhães, Thiago Nilton A. Pereira, Janice Muriel‐Cunha, Alberto Akama

**Affiliations:** ^1^ Museu Paraense Emílio Goeldi, Programa de Pós‐Graduação em Biodiversidade e Evolução Belém Pará Brazil; ^2^ Universidade Federal do Pará, Campus de Altamira, Faculdade de Medicina, Rua Coronel José Porfírio Altamira Pará Brazil; ^3^ Universidade Federal do Pará/EMBRAPA, Programa de Pós‐graduação em Ecologia Belém Pará Brazil; ^4^ Universidade Federal do Tocantins, Laboratório de Ictiologia Sistemática Porto Nacional Tocantins Brazil; ^5^ Universidade Federal do Pará, Programa de Pós‐graduação em Biologia Ambiental Bragança Pará Brazil

**Keywords:** Cichlinae, neuroanatomy, Neuroecology, rapid streams, Rheophilic species

## Abstract

*Teleocichla* comprises small cichlids that inhabit the rapid streams of Amazonian rivers; however, there has been limited research on their encephalon morphology. This study examined the neuroanatomy of four species, focusing on volumetric measurements of their encephalon subregions, and providing a histological description of the encephalon of *T. monogramma*. Our findings reveal that the *tectum mesencephali* and *torus semicircularis* have the highest relative volume across all species examined, followed closely by the telencephalon and ventral diencephalon. The *tectum mesencephali* in some species of *Teleocichla* reached the highest recorded values for neotropical cichlids. The encephalic mass to body mass ratio calculated for the investigated species indicates a level of encephalization similar to that typically observed in taxa with smaller adult body sizes. We assert that these morphological similarities, alongside the pronounced volume of their visual centers, are indicative of their biology, diurnal habits and habitat preferences in complex environments such as clear‐water rapid streams. This research significantly enhances our understanding of the morphological diversity of the *Teleocichla* encephalon and establishes new histological insights into the encephalon of the neotropical cichlids.

## 1 INTRODUCTION

Cichlids are outstanding models for neurobiology and comparative biology due to their remarkable diversity in behavior, diet, and social structure. Their encephalic variation and the evolutionary aspects of their central nervous system have been extensively studied, particularly in species from Africa's Great Lakes (Victoria, Tanganyika, and Malawi), where they have undergone an extraordinary adaptive radiation (Huber et al., 1997; Kornfield & Smith, 2000; Kotrschal et al., 1998; Maruska & Fernald, 2018; van Staaden et al., 1995; York et al., 2019).

Neuroanatomical data can be applied to research from various perspectives, including ecological (Huber et al., 1997; Kotrschal et al., 1998; Shumway, 2010), behavioral (York et al., 2019) and phylogenetic applications. In this context, it is important to highlight recent studies on different groups of Neotropical fish (Characidae: Pereira & Castro, 2016; Callichthyidae: Pupo & Britto, 2018; Heptapteridae: Abrahão et al., 2018a; Pseudopimelodidae: Abrahão et al., 2018b; Cichlinae: de Oliveira & da Graça, 2024).

Recent studies on Neotropical cichlids reveal a new perspective on encephalic diversity within Cichlinae, particularly among the Geophagini (de Oliveira & da Graça, 2020, 2024). These studies propose neuroanatomical characters for phylogenetic inference and suggest potential adaptive convergences related to the gustatory centers in the dorsal *medulla*. This is evident in taxa like *Geophagus* and *Retroculus*, which use a substrate‐sifting feeding strategy and have complex gustatory systems located in the dorsal *medulla*. In contrast, carnivorous groups like *Acaronia*, *Saxatilia*, and *Cichla* exhibit reduced taste centers.

The dorsal *medulla* of cichlids, with regard to the gustatory centers, is a structure with high morphological plasticity (York et al., 2019). In Cichlinae, variations in feeding strategies may play a significant role in the plasticity of the dorsal *medulla*, as Neotropical cichlids possess adaptations related to the buccal apparatus and the oropharyngeal region, which are closely associated with feeding strategies or mouth brooding (Arbour & López‐Fernández, 2016; López‐Fernández et al., 2012; Waltzek et al., 2003).

Additionally, there are indications that different environmental conditions may influence the encephalic morphology of this group (de Oliveira & da Graça, 2024), which demonstrates the potential of neuroanatomical approaches, in an additive way, for a better understanding of the evolutionary/adaptive aspects of Cichlinae.

The limited availability of neuroanatomical data for Cichlinae hinders detailed discussions, particularly considering the subfamily comprises over 500 valid species across the Neotropics (Fricke et al., 2024). This group encompasses taxa that inhabit complex environments, such as the rapids of large Amazonian rivers, where specialized fish have adapted to intense hydrological dynamics (Fitzgerald et al., 2018).

Among the neotropical cichlids specialized in living in rapids, the genus *Teleocichla* stands out, including small‐sized species (on average 76 mm) (Kullander, 1988) and predominantly inhabiting lotic environments associated with rocks and sandy bottoms in large Amazonian rivers (Kullander, 1988; Varella et al., 2016, 2023; Varella & Moreira, 2013). All species within this genus have an elongated body shape, a small mouth likely adapted for an invertivorous diet, and other morphological and behavioral adaptations for survival in these environments. These adaptations include highly developed eyes, a head‐up body position during active periods, and fins modified to support the body on the substrate (Alberto Akama, 2024, pers. comm.).

Recent research has examined the neuroanatomy of *Teleocichla* specifically *Teleocichla proselytus* Kullander, 1988 (de Oliveira & da Graça, 2024), but detailed studies on other species and their microanatomy are limited. This hinders our understanding of the evolution and neuroecology of *Teleocichla* and Neotropical cichlids.

In this study, we provide new data on the gross encephalon anatomy of *Teleocichla* and compare different aspects of the encephalon morphology of the species based on the biology of the group, other species of Geophagini, environment, and body size, contextualized with the availability of data in the literature from functionally well‐known encephalon subregions. Additionally, we describe for the first time the histology of the encephalon of *T. monogramma* as a basis for comparative studies between *Teleocichla* and Cichlinae.

## 2 MATERIALS AND METHODS

### 2.1 Sampling

Specimens of *Teleocichla centrarchus* Kullander, 1988 (*N* = 3), *T. monogramma* (*N* = 23; 10 males and 13 females), and *T. gephyrogramma* Kullander, 1988 (N = 3) were collected in August and September 2023 during an expedition in the rapid streams of the Xingu River (reference point coordinates 03°42′43.72″S 52°27′53.10″W). Sampling was performed by snorkeling with small handmade nets (2 × 2 mm mesh) in sandy‐bottomed rapid streams composed of quaternary ironstone formations (Freire et al., 2022), locally referred to as “mocororô”. In addition, encephalon data for *T. proselytus*, recorded in a previous study (de Oliveira & da Graça, 2024), were included for comparative purposes (see Table 2).

All individuals used in this study measured between 31 and 46 mm in standard length. Sex was determined by macroscopic examination of the gonads. Morphometric data were obtained using an analytical balance (precision: 0.001 g) and digital calipers (precision: 0.01 mm), following the methodology of Kullander (1988).

### 2.2 Ethical considerations

Specimens of *Teleocichla* were collected and transported in accordance with Brazilian regulations under SISBIO collection license number #89240. No experiments were conducted on live animals. After collection, individuals were acclimated in buckets and euthanized using a lethal dose of clove oil (0.20 mL), following the protocol established by Fernandes et al. (2016). Subsequently, specimens were fixed in 10% buffered formalin for at least 48 h, rinsed in running water, and preserved in 70% ethanol.

### 2.3 Encephalon data collection and analysis

The specimens were craniotomized using a stereomicroscope and ophthalmic surgical instruments, including fine‐tipped forceps, Vannas scissors, rat‐tooth forceps, scalpel blades, and insulin needles.

The nomenclature of the principal subregions follows Meek and Nieuwenhuys (1998), and the internal subregions of the encephalon, the nomenclature follows Northcutt (2006). To compare the external morphology with previously published data, the main subdivisions adopted in this study are as follows: telencephalon, mesencephalon (*tectum mesencephali* plus *torus semicircularis*), diencephalon (ventral part), rhombencephalon (only the *corpus cerebelli* and *eminentia granularis* were measured together). Although the *medulla oblongata* is part of the rhombencephalon, its volumetric estimation was not feasible due to the lack of precise anatomical boundaries in the analyzed sample.

The dorsal, ventral and lateral images were obtained using a calibrated auto‐stacking multi‐focus stereomicroscope with an attached camera (Leica M205A) operated via Leica Application Suite‐LAS software version 4.1.0. Images were acquired based on the median line formed by the cranio‐caudal axis of the body.

Whole encephalons were immersed in a 70% alcohol solution. All images were analyzed and processed by a single person (RL‐R).

**FIGURE 1:**
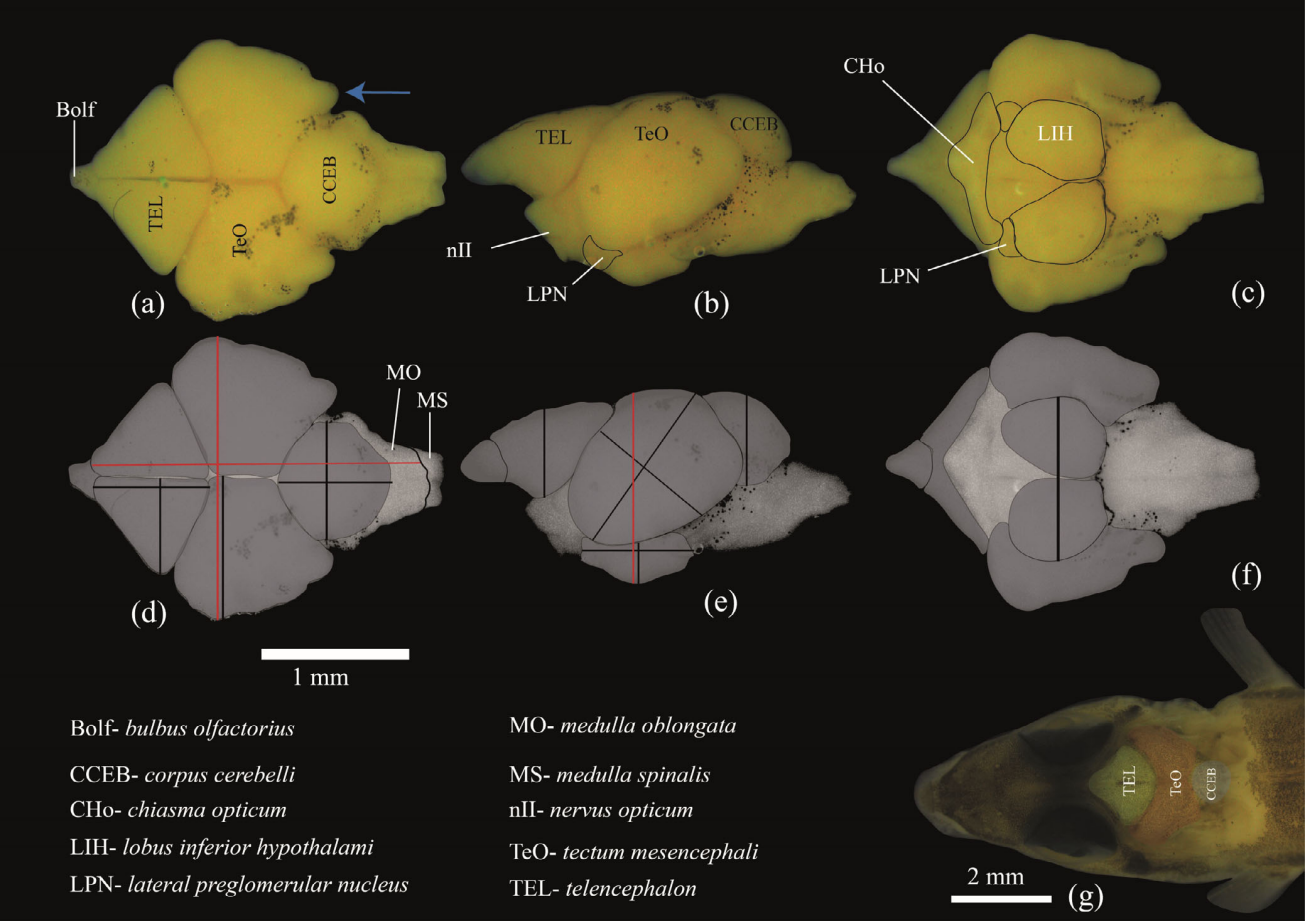
Encephalon of *Teleocichla monogramma*. Photographs showing different views of the encephalon: Dorsal (a), lateral (b), and ventral (c). The blue arrow indicates the caudal extension of the TeO. Schematic drawings of measurements used for volumetric conversion of the main subregions of the encephalon (d–f). The red lines show the linear measurements to estimate the total encephalon volume, while the black lines indicate the linear measurements to estimate the volume of each corresponding sub‐region. Highlighting the anatomical position of the encephalon in the skull, in dorsal view (g).

Fifteen linear measurements were taken, comprising length, height, and width of the encephalon and its main subdivisions (telencephalon, *tectum mesencephali* + *torus semicircularis*, diencephalon, *corpus cerebelli* + *eminentia granularis*). The total length of the encephalon of the species *T*. *centrarchus*, *T*. *gephyrogramma* and *T*. *monogramma* was measured from a parallel line starting from the most rostral portion of the telencephalon to the most caudal portion of the *medulla oblongata*. The telencephalon and *tectum mesencephali* were measured in only one hemisphere (left) and had their volume values doubled (Figure 1). The respective calculated values of the linear measurements are available in Table S1.

The total encephalon volume was calculated based on the total length of the encephalon, as described above, the height (in lateral view, from the most anterior portion of the diencephalon to the dorsal portion of the *tectum mesencephali*) and width (in dorsal view, from the right and left extremities of the *tectum mesencephali*) (Figure 1). The estimated volumes for *T*. *proselytus* were obtained from de Oliveira and da Graça (2024). The measurements were taken using ImageJ (v. 1.53t). Subsequently, the linear measurements were converted into volume using an ellipsoid model to estimate the volume of each sub‐region and the total encephalon volume, an adequate method in neuroanatomical studies (White & Brown, 2015).

Volumetric conversion from the linear measurements of the main subregions of the encephalon was performed according to the following formula: *V* = ⅙ *π*L*W*H where the acronyms represent, respectively, *V* = volume, *L* = length, *W* = width, and *H* = height (Shumway, 2010).

For the description, the volume of each subregion was expressed as a percentage of the total encephalon volume, calculated by dividing the volume of the subregion by the total encephalon volume.

To test whether there are differences in the means of total encephalon volume and the volumes of encephalon subregions between male and female *T. monogramma*, a linear model (LM) was implemented. The variable sex was used as the predictor, and the total encephalon volume and the volumes of the subregions were the response variables. We did not test the influence of sexual dimorphism for the other species, due to the low sample size. Values of *p* ≤ 0.05 were considered significant. Statistical analyses were performed using R Studio (R Core Team, 2022).

### 2.4 Histological procedures

The encephalons of three *Teleocichla monogramma* specimens were prepared for histological characterization. The whole encephalons were embedded in 4% agarose blocks and sectioned in the transverse plane using a vibratome (Leica, VT1000 S) set to a thickness of 80 μm. The sections were then mounted on gelatinized slides and counterstained with cresyl violet using the following protocol: (1) two baths in distilled water; (2) immersion in cresyl violet solution for 10 min; (3) immersion in a solution of 70% alcohol +27% acetic acid for 30 s; (4) immersion in cresyl violet for 5 min; (5) immersion in a solution of 70% alcohol +27% acetic acid for 10 s; (6) immersion in 95% alcohol solution for 2 min; (7) immersion in absolute alcohol for 2 min; (8) immersion in xylene for 10 min; (9) mounting with Canada balsam. The sections were photographed using an optical microscope with a 10x objective lens (Nikon Eclipse CI with motorized stage).

### 2.5 Nomenclature

Characterization and internal tissue nomenclature were based on a 2D reference atlas for *Oreochromis mossambicus*—Cichlidae (Pepels et al., 2002), *Danio rerio*—Ciprinidae (Wullimann et al. 1996), *Dicentrarchus labrax*—Moronidae (Cerdá‐Reverter et al., 2001a: telencephalon; Cerdá‐Reverter et al., 2001b: diencephalon; Cerdá‐Reverter et al., 2008: mesencephalon and rhombencephalon). The illustrations in the sections and the editing of images were done using image editors.

The abbreviations for the main subregions of the encephalon used in this study are as follows: Adt: *area dorsalis telencephali*, Avt: *area ventralis telencephali*, BOlf: *bulbus olfactorius*, CCEB: *corpus cerebelli*, CCe: *crista cerebelaris*, CCg: granular layer of *corpus cerebelli*, CCm: molecular layer of *corpus cerebelli*, CM: *corpus mamillare*, DC‐1: central layer 1 of Adt, DC‐2: central layer 2 of Adt, Die: diencephalon, DL‐d: lateral dorsal layer, dorsal part of the Adt, DL‐v: lateral dorsal layer, ventral part of the Adt, DM: medial dorsal layer of the Adt, DTN: dorsal tegmental nucleus, E: *nucleus ependimal*, EG: *eminentia granularis*, EXLolf: glomerular layer of the *bulbus olfactorius*, Flm: *fasciculus longitudinalis medialis*, GLolf: granular layer of *bulbus olfactorius*, h: horizontal commissure fibers, HB: *habenulla*, HC: caudal periventricular zone of the hypothalamus, Hv: periventricular ventral zone of the hypothalamus, Lca: *lobus caudalis cerebelli*, LF: *lobus facialis*, LLF: lateral longitudinal fascicle, LPN: *lateral preglomerular nucleus*, LX: *lobus vagi*, Ma: Mautner's axon, MO: *medulla oblongata*, MON: medial octavolateral nucleus, MS: *medulla spinalis*, Namb: *nucleus ambiguus*, NG: *nucleus glomerular*, NIII: *nervus oculomotor*, NLD: diffuse nucleus of the *lobus inferior hypothalami*, NT: *nucleus taenia*, NX: *nervus vagus*, Ot: *tractus opticum*, Pcom: posterior commissure, PGm: medial preglomerular nucleus, POA: pre‐optical area, RF: reticular formation, RLDie: lateral recess of the diencephalic ventricle, RV: ventricle of rhombencephalon, SE: *sulcus externus*, SL: *sulcus limitans*, SPV: *stratum periventriculare*, SY: *sulcus ipsilyformis*, TEL: telencephalon, TeO: *tectum mesencephali*, TL: *torus longitudinalis*, TLa: *torus lateralis*, TS: *torus semicircularis*, Valce: *valvula cerebelli*, VDi: ventricle of diencephalon, VMs: ventricle of mesencephalon, Vp: *nuclei pos‐commissural*, *Vtel*: *ventricle* of telencephalon, VV‐ *nuclei ventral*, Vvn: ventral area of Avt.

## 3 RESULTS

**FIGURE 2:**
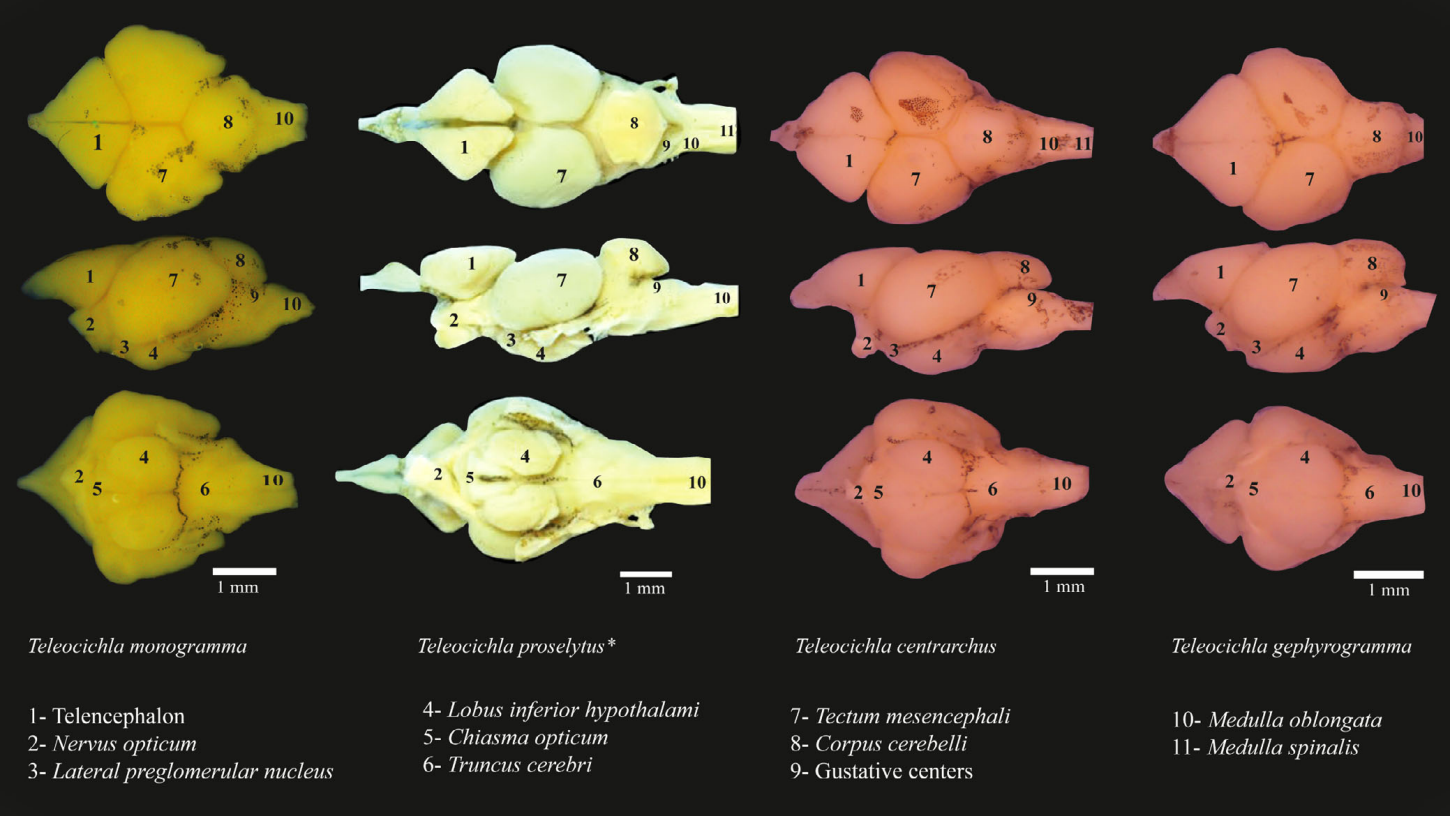
Encephalons of the analyzed species of *Teleocichla* arranged in dorsal, lateral, and ventral views. The image of *T*. *proselytus* is from de Oliveira & da Graça, 2024 (Springer Nature License number 6142041292517).

In total, 23 individuals of *Teleocichla monogramma* (10 males and 13 females), together with three specimens each of *T. gephyrogramma* and *T. centrarchus*, were analyzed, with their measurements summarized in Table 2. For comparative purposes, encephalon data previously reported for *T. proselytus* (de Oliveira & da Graça, 2024) were also incorporated (Figure 2). No significant differences were detected between males and females of *T. monogramma* regarding encephalon mass and the estimated volumes of the analyzed subregions (Table 1). Consequently, the entire sample (*N* = 23) was considered as a single group for the anatomical characterization of the encephalon.

**TABLE 1:** Linear model results of total encephalon volume and encephalon subregions in comparison between males and females.

Variables	*F*‐statistic	df	*p*‐value
Encephalon volume	0.0002965	21	0.9864
Telencephalon	0.009862	21	0.9218
*Tectum mesencephali* + *torus semicircularis*	0.3988	21	0.5345
Diencephalon	1.176	21	0.2905
*Corpus cerebelli* + *eminentia granularis*	0.04968	21	0.8258

Abbreviation: df, degrees of freedom.

The encephalon of *T*. *monogramma* has a total average volume of 3.85 ± 1.19 mm^3^ (range 2.36–6.70 mm^3^); for *T*. *gephyrogramma*, the recorded average volume was 12.28 ± 3.08 mm^3^ (range 10.19–15.82 mm^3^); for *T*. *centrarchus*, the recorded average volume was 13.14 ± 2.34 mm^3^ (range 10.91–15.58 mm^3^), while for *T*. *proselytus*, since only one specimen was analyzed, the recorded total encephalon volume was 30.17 mm^3^ (Tables 2 and S2).

**TABLE 2:** Measurements and estimated volumes of the encephalons and subregions of the analyzed specimens.

Measurements	*Teleocichla centrarchus* (*N* = 3)			*Teleocichla gephyrogramma* (*N* = 3)			*Teleocichla monogramma* (*N* = 23)			Teleocichla proselytus* (*N* = 1)		
	Range	Mean	SD	Range	Mean	SD	Range	Mean	SD	Range	Mean	SD
SL (mm)	30.13–36.56	32.70	3.39	27.46–36.26	31.01	4.63	34.3–48.4	39.81	4.28	16.85	–	–
HL (mm)	8.25–9.15	8.65	0.45	7.16–9.01	7.90	0.97	8.1–12	9.92	1.06	–	–	–
BM (g)	0.25–0.5	0.35	0.13	0.24–0.55	0.36	0.16	0.38–1.15	0.68	0.22	–	–	–
EM (g)	0.0064–0.0095	0.007	0.001	0.007–0.0097	0.007	0.001	0.0083–0.0207	0.014	0.003	–	–	–
TLE (mm)	3.65–4.14	3.85	0.26	3.55–4.08	3.75	0.29	2.18–3.32	2.62	0.29	–	–	–
EV (mm^3^)	10.91–15.58	13.14	2.34	10.19–15.82	12.28	3.08	2.36–6.7	3.85	1.19	30.17	–	–
CCEB (mm^3^)	0.56–0.68	0.63	0.06	0.48–1.1	0.78	0.31	0.10–0.49	0.21	0.09	1.46	–	–
TEO (mm^3^)	3.36–5.89	4.67	1.27	3.3–5.31	4.16	1.04	0.85–2.16	1.38	0.39	7.36	–	–
DIE (mm^3^)	0.74–0.99	0.91	0.14	0.65–1.28	0.91	0.33	0.11–0.39	0.23	0.07	2.12	–	–
TEL (mm^3^)	1.56–1.88	1.77	0.18	1.51–2.03	1.73	0.27	0.21–0.83	0.45	0.19	2.89	–	–
*Percentages of HL*												
Ratio (TLE/HL) (%)	43.87–45.25	44.45	0.007	45.28–50.70	47.69	0.02	22.46–31.48	26.48	0.02	–	–	–
*Percentages of TLE*												
Ratio (TWE/TLE) (%)	77.54–84.84	80.34	0.03	74.66–81.69	78.10	0.03	71.69–83.64	77.78	0.03	–	–	–
*Percentages of BM*												
Ratio (EM/BM) (%)	1.90–2.63	2.36	0.4	1.76–2.46	2.19	0.37	1.39–2.58	2.06	0.30	–	–	–
*Percentages of total encephalon volume*												
CCEB (%)	4.35–5.11	4.81	0.40	4.7–7.02	6.22	1.31	4.03–7.31	5.45	0.82	4.8	–	–
TeO (%)	30.83–37.81	35.12	3.75	32.33–35.76	33.88	1.74	27.61–43.98	36.14	3.22	24.4	–	–
DIE (%)	5.72–9.06	7.04	1.78	6.4–8.09	7.25	0.85	4.56–7.84	6.10	0.94	7	–	–
TEL (%)	12.1–14.35	13.58	1.28	12.85–15.32	14.32	1.30	8.21–14.85	11.39	1.90	9.6	–	–

*Note*: Values expressed are proportional to the total encephalon volume, head length or encephalon length. * data from de Oliveira and da Graça (2024).

Abbreviations: BM, body mass; CCEB, volume of *corpus cerebelli* + *eminentia granularis*; DIE, volume of diencephalon; EM, encephalon mass; EV, encephalon volume; HL, head length; SL, standard length; TEL, volume of telencephalon; TeO, volume of *tectum mesencephali* + *torus semicircularis*; TLE, total encephalon length; TWE, total encephalon width. The data to estimate the ratio (TLE/HL) and (TWE/TLE) are available in the Table S1.

On average, encephalon's mass corresponds to 2.06% of body mass for *T*. *monogramma*; 2.36% for *T*. *centrarchus* and 2.19% for *T*. *gephyrogramma*. The total length of the encephalon is 26.46% of the total length of the head for *T*. *monogramma*; 44.45% for *T*. *centrarchus* and 47.69% for *T*. *gephyrogramma* (Table 2).

Variations in the shape and volume of the subregions for all species will be detailed and discussed throughout this study. The cranial nerves reflect the general pattern observed in teleosts.

### 3.1 Gross encephalon anatomy of the *Teleocichla* species and histological encephalon description of *Teleocichla monogramma*

#### 3.1.1 The *bulbus olfactorius* (BOlf)

**FIGURE 3:**
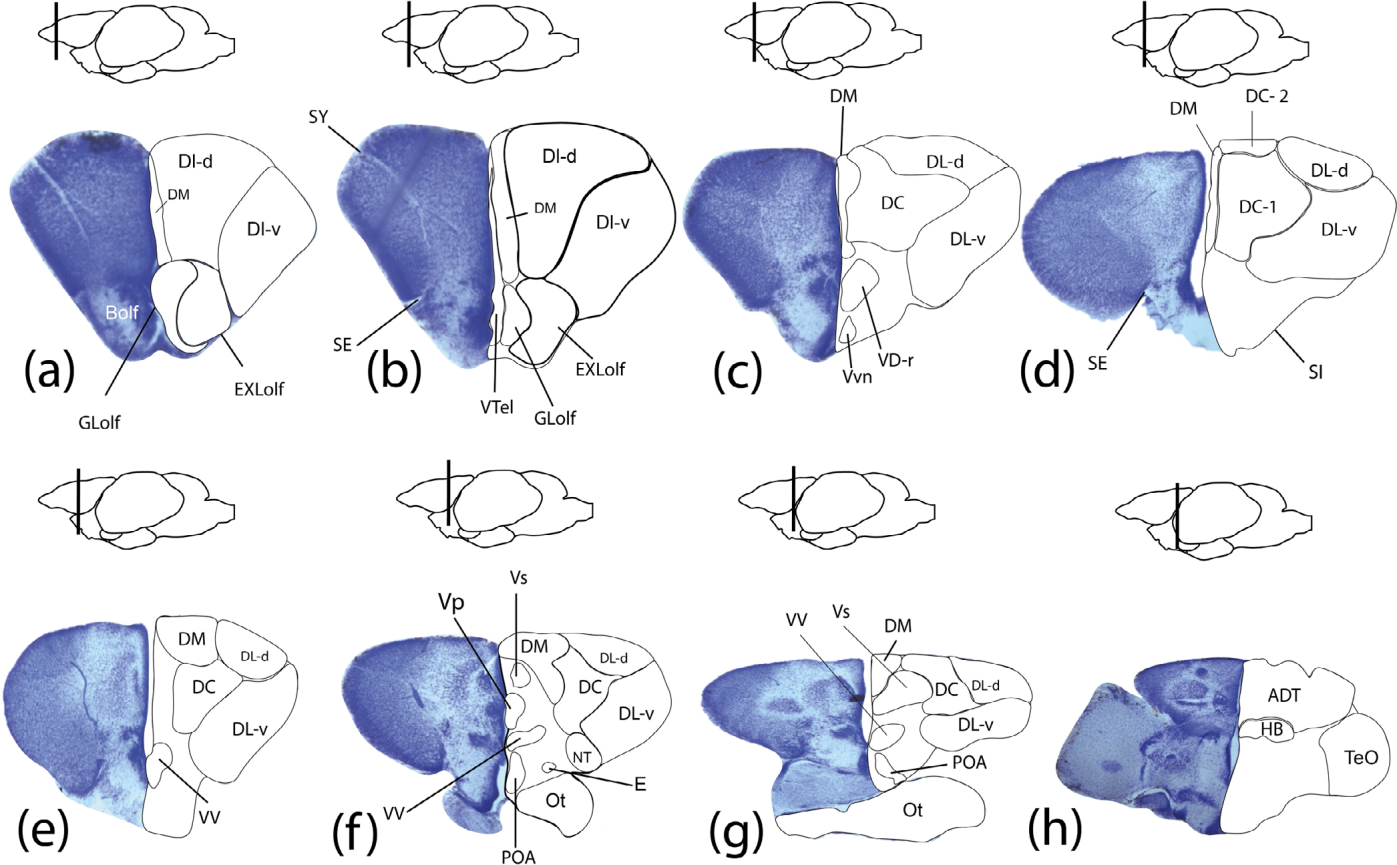
Photomicrographs of eight transverse sections of the telencephalon of *Teleocichla monogramma*, along with the corresponding drawings of the equivalent areas. Sections are arranged from (a) to (h) in a rostral‐caudal direction. The distances between sections are not indicated. All sections were photographed using a 10× lens, though they are not all represented at the same scale. Section thickness is 80 μm. Sections stained with cresyl violet.

In all observed species, the BOlf is sessile and can be observed more rostrally to the insertion of the telencephalon. In histological sections for *T*. *monogramma*, the BOlf can be observed more rostrally to the insertion of the telencephalon. In this case, the BOlf is sessile. The BOlf is connected with the Avt and, in *T. monogramma*, it exhibits two large identifiable layers (Figure 3a) with distinct cell densities. The innermost layer, the granular layer of the BOlf (Glolf), features concentric and densely packed cells. In contrast the outermost layer, the glomerular layer of the BOlf (EXLolf), contains regions with spaced cells and, in some areas, densely packed cells.

#### 3.1.2 Telencephalon

It is the most anterior subregion of the encephalon and is organized into two hemispheres separated by a longitudinal fissure. In *T*. *monogramma*, *T*. *gephyrogramma* and *T*. *centrarchus* this fissure is narrower than in *T*. *proselytus*. In all examined species, the telencephalon is a well‐developed structure relative to the other subregions, though not hypertrophied. The telencephalon of *Teleocichla* is triangle‐shaped in dorsal view, with the surface free of prominences indicative of gross pallial zones, except in *T*. *proselytus*. It is the second largest subregion of the encephalon, with the highest recorded relative volume values for *T*. *gephyrogramma* (14.32 ± 1.30%), followed by *T*. *centrarchus* (13.58 ± 1.28%) and *T*. *monogramma* (11.36 ± 1.91%) (Table 2).

The telencephalon of fish is divided into two main regions: the *area dorsalis telencephali* (Adt) and the *area ventralis telencephali* (Avt). These regions can be further subdivided based on differences in cellular organization.

In *T. monogramma* we observed the presence of three pallial delimitation zones bordering the Adt and Avt: the *sulcus ipsilyformis* (SY) (Figure 3b), the *sulcus externus* (SE) (Figure 3b,d) and the *sulcus limitans* (Sl) (Figure 3d). Dorsally, SY can only be seen more conspicuously in histological sections.

As shown in histological sections (Figure 3h), the Adt slightly overlaps the anterior portion of the *tectum mesencephali*.

In the Adt, three distinct pallial regions can be distinguished based on cell size and density. These regions include the medial dorsal region, which shows a progressive concentration of cells in a rostral and medial orientation; the lateral dorsal part, which is divided into Dl‐d and Dl‐v; and the central dorsal region, which is divided into DC‐1 and DC‐2 (Figure 3f).

In the Avt, three main groups of cells organized into clusters, which constitute the nuclei, were identified: the ventral nucleus (Vv), the supra‐commissural nucleus (Vs), and the post‐commissural nucleus (Vp) (Figure 3f). The ependymal nucleus (E) was observed in the more basal portion of the Avt, in the medial‐lateral part of the POA, situated rostrally near the *sulcus externus* (Figure 3d), in close contact with what we tentatively identify as the *nucleus taenia* (NT) (Figure 3f).

#### 3.1.3 Mesencephalon

It comprises an important subregion of the encephalon, serving as a primary center for processing visual and auditory information and lateral line stimuli. It primarily comprises the *tectum mesencephali*, the *torus semicircularis*, and the *ventral tegmentum* (Northmore, 2011).

#### 3.1.4 *Tectum mesencephali* + *torus semicircularis* (TeO)

The TeO is markedly well‐developed in comparison to other encephalic subregions and represents the largest volumetric component of the encephalon across all analyzed species (Tables 2 and S2). Among the *Teleocichla* species investigated from the Xingu River, the TeO differs notably from that of *T*. *proselytus* exhibiting a more oval shape and a comparatively lower relative volume (Figure 2 and Table 2). In contrast, the TeO in *T*. *monogramma*, *T*. *centrarchus*, and *T*. *gephyrogramma* displays a slight lateral expansion toward the rostral direction. Notably, *T. monogramma* can be morphologically distinguished by a caudally directed lateral prominence of the TeO that extends toward the medial region of the *corpus cerebelli*. Quantitatively, the TeO width corresponds to 77.78% of the total encephalon length in *T. monogramma*, 80.34% in *T. centrarchus*, and 78.10% in *T. gephyrogramma* (Table 2).

In fish, especially actinopterygians, TeO is a paired structure with a laminated cytoarchitecture, mainly composed of six layers, the outermost of which are: *stratum marginale*, *stratum opticum*, *stratum fibrosum et griseum superficiale*; the innermost: *stratum griseum centrale*, *stratum album centrale* and *stratum periventriculare* with different functions (e.g., visual, multimodal or motor centers) (Northmore, 2011).

**FIGURE 4:**
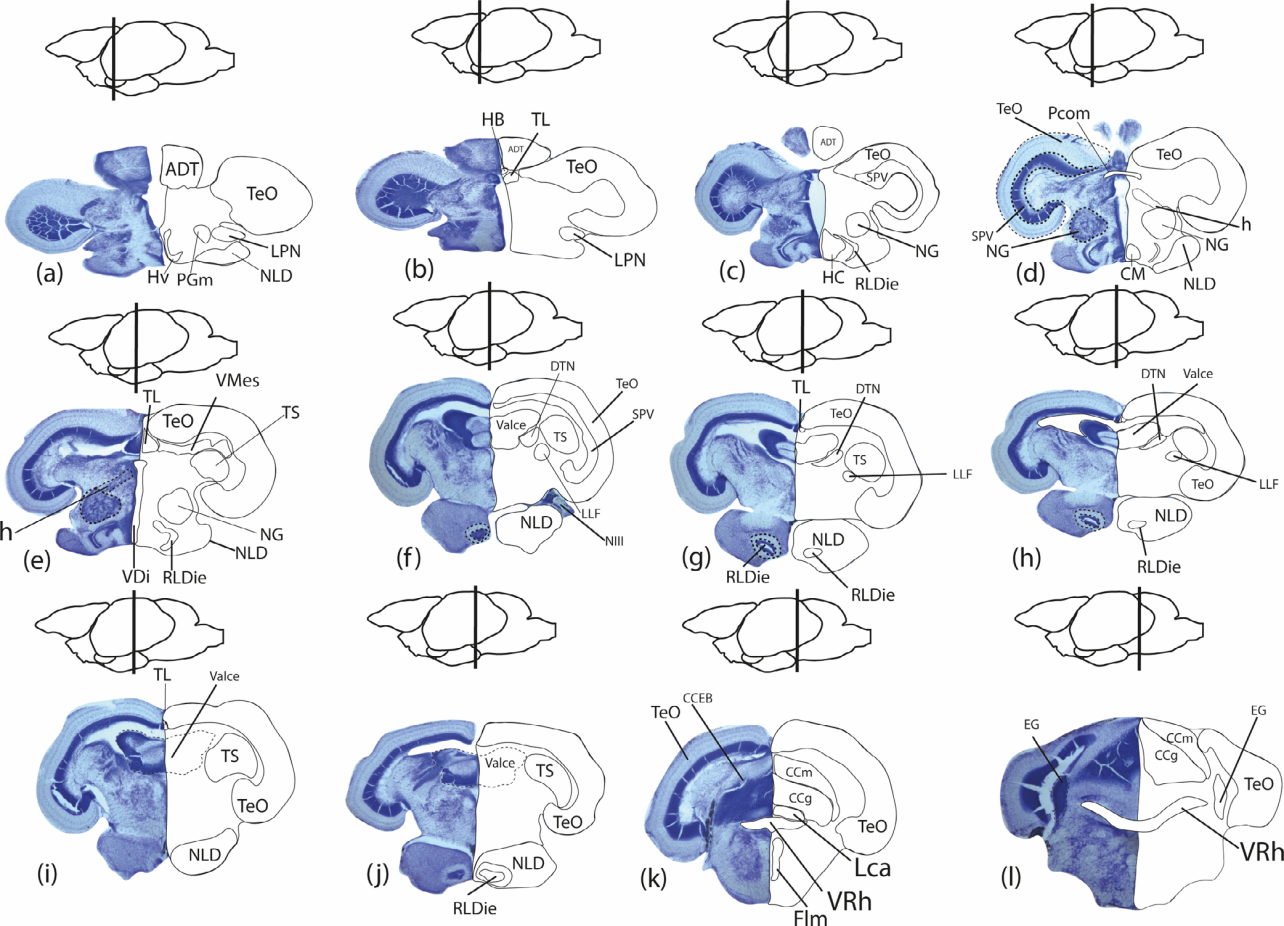
Photomicrographs of 12 transverse sections of the mesencephalon and diencephalon of *Teleocichla monogramma*. Sections are arranged from (a) to (l) in a rostral–caudal direction. Graphical conventions as in Figure 3.

All six layers are visible in *T*. *monogramma*. The *stratum periventriculare* (SPV) is the most conspicuous layer in the TeO and is characterized by a dense accumulation of neural cells prominently marked by Nissl staining. In *T. monogramma*, the SPV is widening at its initial insertion in a dorso‐rostral direction (Figure 4c–k). As it progresses caudally, the SPV also widens in the ventral portion (Figure 4f–k). Caudally, the *stratum periventriculare* is in contact with the molecular layer of the *corpus cerebelli* and the *eminentia granularis* (Figure 4l).

The *torus longitudinalis* in *T. monogramma* is qualitatively more developed in the rostral direction and reduced in size in the caudal direction.

#### 3.1.5 Diencephalon

**FIGURE 5:**
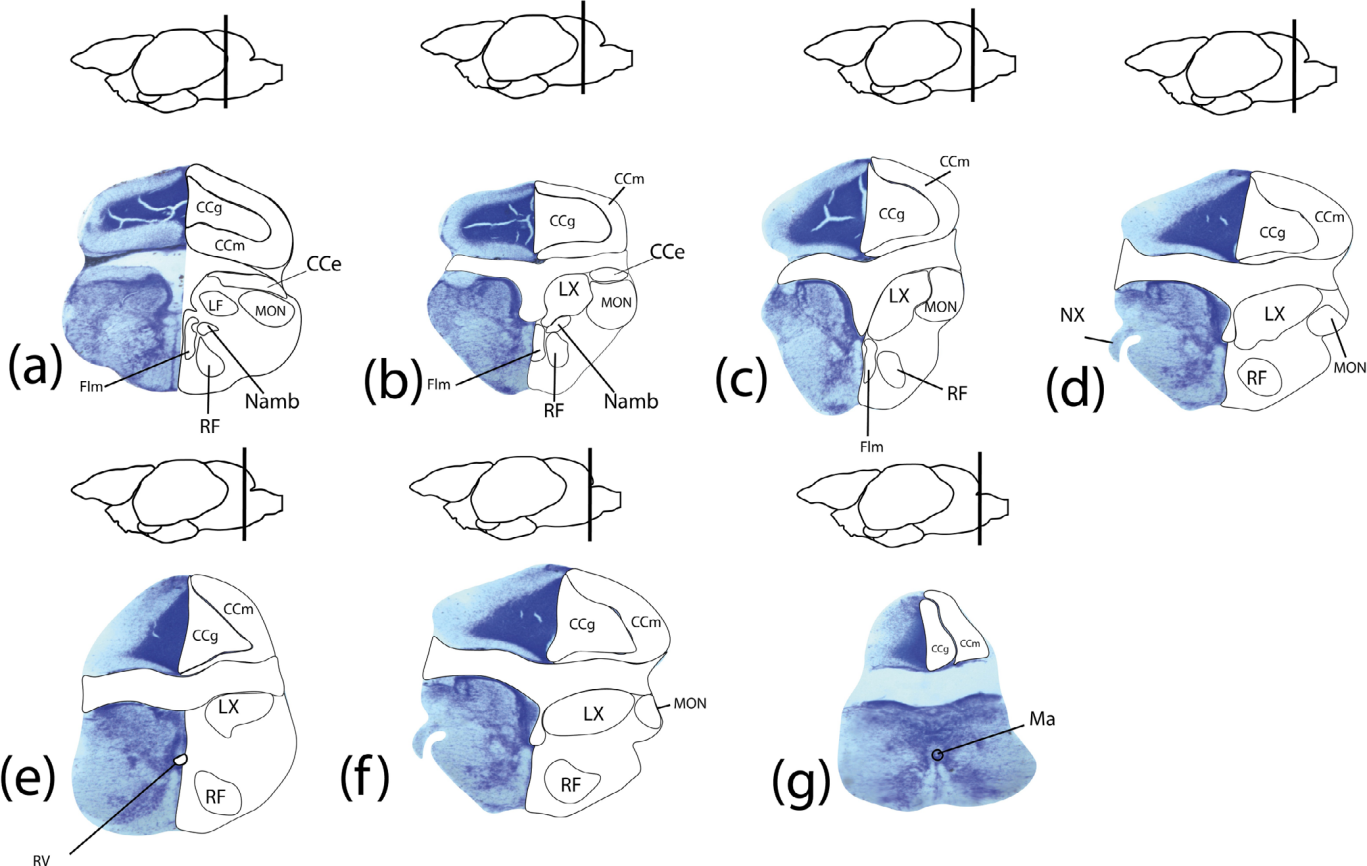
Boxplot proportions of main subdivisions of the encephalons of *Teleocichla centrarchus*, *T. gephyrogramma* and *T. monogramma*. CCBE, *corpus cerebelli*; DIE, diencephalon; TEL, telencephalon; TeO, *tectum mesencephali* + *torus semicircularis*.

In all examined species, except for *T*. *gephyrogramma*, the diencephalon represents the third largest encephalic subregion in terms of average relative volume (Table 2 and Figure 5).

The pineal gland was either inconspicuous or damaged during the dissection process in all specimens. In most teleosts, the *habenula* is a paired structure, typically small and located in the dorsal region of the epithalamus, and it can occasionally be visualized from a dorsal perspective. The morphology of the diencephalon shows minimal variations between species, as does the relative volume; this structure is not conspicuous due to the contact between the more rostral margin of the telencephalon and the initial limit of the dorsal region of the *tectum mesencephali*, which is more dorsally truncated (Figure 2).

However, in histological sections of the encephalon of *T. monogramma* the *habenula* is visualized in the posterior portion of the *area dorsalis telencephali* just above the *torus longitudinalis* (Figure 4b).

During the removal of the encephalon, it was not possible to preserve the hypophysis. In all examined species, the hypophysis lies in close contact with the *chiasma opticum* and is positioned within a depressed region at the base of the neurocranium. The *saccus vasculosus* was consistently reduced and not conspicuous in lateral view. Due to the delicate nature of the dissection, this structure was also frequently compromised. When preserved, the *saccus vasculosus* was located in the most rostral portion of the hypothalamus, specifically in the central region of the *lobus inferior hypothalami*.

In histological sections of the diencephalon of *T*. *monogramma*, the *nucleus glomerular* (NG) is prominently observed in the medial region between the thalamic area and the lower lobe of the diencephalon. This nucleus is large, rounded in shape, and characterized by a heterogeneous population of cells of varying sizes, which are dispersed throughout without forming distinct layers (Figure 4c–e).

In the inferior diencephalon lobe, we observed the presence of the lateral recess of the diencephalic ventricle—RLDie, composed of two layers of cells: a dense layer surrounding the ventricle and a sparse layer with a few cells (Figure 4g). The NLD is composed of small, dense, and uniform cells.

#### 3.1.6 Rhombencephalon

This region is in the most caudal portion of the teleost encephalon, bounded in the rostrocaudal plane by the *tegmentum mesencephali* and the *medulla spinalis*. In the species examined, the *eminentia granularis* can be observed in the dorsal view of the most posterior mid‐lateral portion of the *corpus cerebelli* (CCEB). In all examined species, the *corpus cerebelli* has a semi‐oval shape with a projection extending caudally to reach the medial portion of the dorsal *medulla*, nearly at the boundary with the initial insertion of the *medulla spinalis*.

Among the investigated species, the CCEB shows few variations in volume, being the fourth largest structure in relative volume of the encephalon. However, qualitatively, there are differences between the species. In *T. gephyrogramma*, in lateral view, the CCEB is higher than in the other species (Figure 2 and Table 2). In *T. centrarchus*, the CCEB exhibits a more elongated shape, and in *T. monogramma*, it presents a more truncated shape, both in lateral view. Unlike in *T. proselytus*, in the other above‐mentioned species, the caudal projection of the CCEB extends beyond the limits of the initial insertion of the gustatory centers, preventing its visualization in dorsal view. Regarding the relative volumes calculated for this subregion, the highest values were recorded for *T. gephyrogramma* (6.22 ± 1.31%), followed by *T. monogramma* (5.45 ± 0.82%), while *T. centrarchus* and *T. proselytus* showed similar values (Table 2).

**FIGURE 6:**
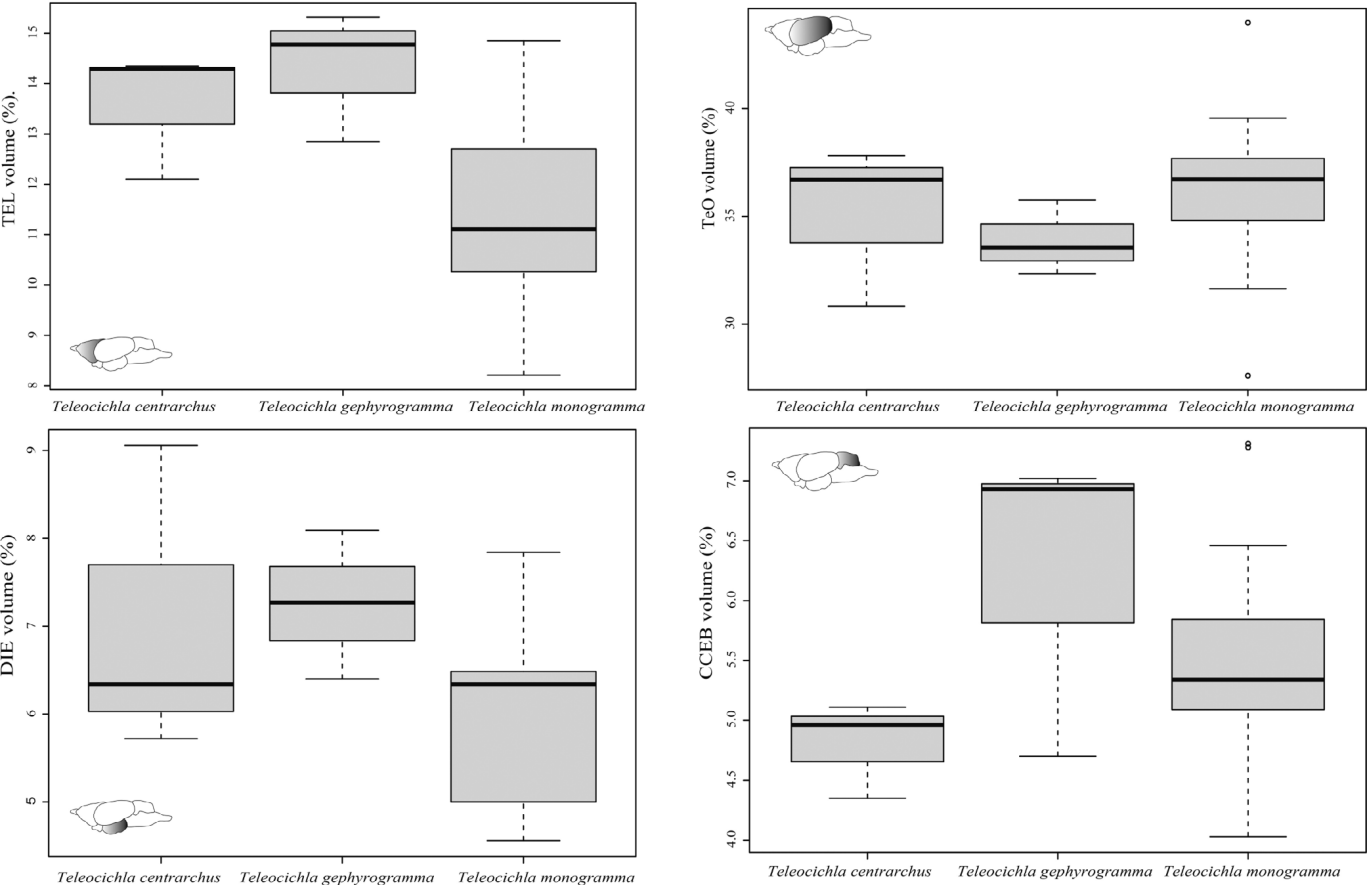
Photomicrographs of seven transverse sections of the rhombencephalon of *Teleocichla monogramma*. Sections are arranged from (a) to (g) in a rostral–caudal direction. Graphical conventions as in Figure 3.

The histology of the CCEB for *T*. *monogramma* shows a two‐layer conformation: the innermost layer (CCg) is qualitatively more prominent and shows dense clusters of neural cells (mostly motor neurons). In contrast the outermost layer (CCm) has spaced neural cells with punctual concentrations of cells along the structure. The contrast between the two layers in the rostrocaudal direction shows a pyramidal conformation (Figure 6c–g).

In the dorsal view, the decussation of the *crista cerebellaris*, the somatosensory centers in the dorsal *medulla*, and the fourth ventricle are not visible, except in *T*. *proselytus* (Figure 2). The taste centers in *T*. *monogramma*, *T*. *gephyrogramma* and *T*. *centrarchus* could not be measured using the ellipsoid method as they are inconspicuous in the dorsal view; they can only be visualized in the lateral view.

The taste centers in *T*. *monogramma*, qualitatively, both the *lobus vagi* and *lobus facialis* are reduced. Histologically, the *lobus facialis* (LF) can be observed immediately after the posterior insertion of the lateral projection of the *tectum mesencephali* (Figure 6a). The LF is positioned peripherally to the ventricle of the rhombencephalon, near the *crista cerebelaris*. The LF has an oval shape and is more developed in the rostral direction, being more conspicuous in a transverse section corresponding to the middle portion of the *corpus cerebelli*. The LF of *T. monogramma* shows a moderate density of neural cells, distributed uniformly.

The *lobus vagi* in *T. monogramma* is developed but lacks prominent features. The LX shows rostral development and is qualitatively more developed than the LF. Nonetheless, there is a moderate concentration of neural cells dorsolaterally (Figure 6b–f).

## 4 DISCUSSION

The generalized morphology of the encephalon of *Teleocichla* sp. (Figure 2) follows the basic plan previously published for the Percomorpha clade (Betancur‐R et al., 2013) according to extensive publications for Cichliformes: Cichlinae—Geophagini (de Oliveira & da Graça, 2020, 2024), African cichlids such as *Haemitilapia oxyrhynchus* (Huber et al., 1997), *Oreochromis mossambicus* (Pepels et al., 2002), *Astatotilapia burtoni* (Maruska & Fernald, 2018); Gobiiformes: *Rhyacichthys aspro* (Rhyacichthyidae) (Bauchot, Diagne, et al., 1989b), *Boleophthalmus pectinirostris* (Oxurdecidae) (Jiang et al., 2023); Perciformes: *Dicentrarchus labrax* (Moronidae) (Cerdá‐Reverter et al., 2001a, 2001b; Cerdá‐Reverter et al., 2008), *Toxotes chatareus* (Toxotidae) (Karoubi et al., 2016), *Ophthalmolycus amberensis* (Zoarcidae) (Lannoo & Eastman, 2006); Beloniformes: *Oryzias latipes* (Adrianichthyidae) (Ishikawa et al., 1999); Cyprinodontiformes: *Austrolebias* (Fernández et al., 2011), *Garcialebias charrua* (Torres‐Pérez et al., 2024) (Rivulidae), *Nothobranchius furzeri* (Nothobranchiidae) (D'Angelo, 2013), Syngnathiformes: *Hippocampus barbouri* (Syngnathidae) (Senarat et al., 2021). Conservatively, this arrangement corresponds to sessile *bulbus olfactorius*, *corpus cerebelli* everted caudally, *torus semicircularis* not conspicuous, and *hypophysis* anchored close to *chiasma opticum*. The encephalon morphology of *Teleocichla* sp. investigated from the Xingu River shows few qualitative differences in gross morphology. These similarities may, in part, be attributed to the species' similar ecological niches and the fact that they were collected from relatively close rapid streams environments in the Volta Grande do Xingu, as has been observed in other teleostean taxa (Kotrschal et al., 1998).

The species of *Teleocichla* investigated here include species that are benthopelagic, diurnal with high visual acuity and inhabit low turbidity, spatially heterogeneous, and complex environments such as the rapid streams of the Xingu River basin (Zuanon, 1999). This species' morphological characteristics and biology may be associated with the high relative volumes observed for the *tectum mesencephali* and the qualitatively reduced *bulbus olfactorius*. In the context of comparative morphology, data from de Oliveira and da Graça (2024) indicate that *Teleocichla* species possess the largest relative volumes of visual centers among Neotropical cichlids. This condition supports the hypothesis of a morphological specialization linked to visual processing, potentially driven by selective pressures associated with the structurally complex and visually demanding habitats of rapid stream environments.

A reduced TeO morphology is commonly found in organisms with low visual acuity or nocturnal habits, such as catfishes, including species of the Trichomycteridae (genus *Ituglanis*) (Rizzato & Bichuette, 2024), Pseudopimelodidae (Abrahão et al., 2018b, 2021; Abrahão & Shibatta, 2015), Callichthyidae (Pupo & Britto, 2018), and Heptapteridae (Abrahão et al., 2018a). High relative volumes of TeO have been widely recorded for visually oriented groups (Kawamura et al., 1981; Lisney & Collin, 2006; Munz & McFarland, 1977), as well as neotropical cichlids (de Oliveira & da Graça, 2020, 2024) and cichlids of the Great African Lakes, especially those belonging to the clade Ectodini (Pollen et al., 2007; Shumway, 2010). These results come from comparative studies considering the relative sizes and cytoarchitectonics of the sensory areas of the encephalon concerning the species's ecological niche. These areas tend to oscillate in development according to the sensory modality to which they are exposed (Ito et al., 2007; Kotrschal et al., 1998).

The lateral projection of the TeO in a caudal direction of *T. monogramma* (Figure 1a), corresponds to an unusual observation for Cichlidae. The contact of the SPV layer with the granular layer of the *eminentia granularis* corresponds to a pattern observed in evolutionary models such as *Danio rerio* (Wullimann et al., 1996) and *Nothobranchius furzeri* (D'Angelo, 2013). We have no evidence to infer whether this arrangement is associated with sensory specialization or adaptive convergence.

In *Teleocichla*, the triangle‐shaped telencephalon, in dorsal view, is a characteristic shared with all analyzed species and other members of Crenicichlinae (e.g., *Biotecus opercularis*) and Apistogrammines (e.g., *Apistogramma trifasciata* and *Taeniacara candidi*), according to recent records (de Oliveira & da Graça, 2024). However, we do not have evidence to state whether this is a specific characteristic of Geophaginii or if there is a relationship with the morphology of the skull. We did not observe conspicuous saliences on the dorsal portion of the telencephalon, which differs from most cichlids. For example, phylogenetically close taxa like *Geophagus sveni* (de Oliveira & da Graça, 2020) and African species commonly used as experimental models, such as *Oreochromis mossambicus* (Simões et al., 2012) and *Astatotilapia burtoni* (Munchrath & Hofmann, 2010), exhibit notable protrusions in this area.

In cichlids, the telencephalon generally shows prominent protrusions on the dorsal portion, allowing for the definition of gross pallial divisions in the *area dorsalis telencephali* (Adt), separated by sulci such as *sulcus ypsilateralis*. In *T. monogramma*, defining the location of subregions in a gross form within the Adt is impossible. For this taxon, exploring this region through histological studies or advanced imaging techniques such as computed tomography using X‐rays or magnetic resonance imaging with contrast is necessary.

The diencephalon of *T. monogramma* presents the subdivisions described by Meek & Nieuwenhuys (1998): pre‐optical area, *epithalamus*, *thalamus*, *hypothalamus*, tuberal region, pre‐tectum and optical tract. In this region, a pronounced *nucleus glomerulosus* or *corpus glomerulosum pars rotunda* was observed (see Figure 4c–e), which comprises a pre‐tectal structure (Wullimann et al., 1991) widely found in acanthopterygians. The NG appears to be associated with mixed sensory functions, such as vision and olfaction, and evolved only in more derived teleost taxa (Sakamoto & Ito, 1982; Yang et al., 2007) and has no homologous region in mammals.

In some taxa with high visual acuity, the NG is highly pronounced, as reported in recent studies for Labridae (Estienne et al., 2024). The NG is present but reduced, in taxa with lower visual acuity, such as some gobiids that live in intertidal zones, like *Boleophthalmus pectinirostris* (Oxudercidae) (Jiang et al., 2023). In African cichlids, this structure is reported and is less developed than in Labridae but more developed than in *B. pectinirostris* (Estienne et al., 2024; Jiang et al., 2023; Yang et al., 2007).

In *Oreochromis mossambicus*, the NG is a component of a descending pathway retinal‐*tectum*‐*diencephalon*‐*tegmentum* (Yang et al., 2007). In cichlids, this structure seems to remain conserved in both African and American lineages, as shown here for *Teleocichla monogramma*. More studies are needed to understand the role of this center, especially neotropical cichlids, which are widely distributed in dynamic and heterogeneous habitats, as well as behavioral issues directly involved with visual acuity, such as parental care.

The taste centers of *T*. *monogramma* are qualitatively reduced; this characteristic is possibly related to the feeding habits of this species, which feeds on small invertebrates and detritus and uses active foraging to collect food items, with no apparent selection of sediment in the oral cavity. In groups that are specialized in selecting items in the oral cavity, such as the genera *Geophagus* and *Satanoperca*, or species with a palatal organ (“mouth taster”) such as some species of catostomids (Doosey & Bart, 2011; Miller & Evans, 1965), the taste centers in the dorsal *medulla* are highly pronounced, with the innervation of structures in the oropharynx by nerves nIX: *nervus glossopharyngeus* and nX: *nervus vagus*.

On average, the encephalon mass of *Teleocichla* analyzed in this study, except *T*. *proselytus*, constitutes around 2% of the total body mass. This is unusual for teleosts. However, there are records for other groups of fish, such as the Osteoglossiformes (Tsuboi, 2021), particularly, the mormyrid *Gnathonemus petersii*, which has a relative encephalic mass of 3% and compared to vertebrates with exceptional encephalization, such as humans at about 2% (Nilsson, 1996). This encephalic mass/body mass ratio indicates encephalization observed in other small‐sized taxa (Bauchot, Ridet, et al., 1989a). In teleosts, in small‐sized groups, there is evidence that encephalon size/body size ratios increase inversely as body size decreases (Bauchot et al., 1989a; Striedter, 2005).

The genera *Teleocichla*, *Apistogramma*, and some species of *Dicrossus*, represent the smallest cichlids occurring in the Neotropical region. However, we have no systematic comparative studies for these genera to understand if these observations represent potential paedomorphosis associated with the CNS or are involved with sensory specializations or other adaptive/evolutionary aspects.

No significant differences were observed between the volumes of the subregions according to the sexual dimorphism of *T. monogramma*. Differences associated with encephalon size in general or subregions due to sexual dimorphism may be related to cognitive or sensory demands, due to different selection pressures in males and females, such as parental care or sexual selection (Garamszegi et al., 2005). In studies for Lake Tanganyika cichlids, Gonzalez‐Voyer et al. (2009) observed that cognitive demands, when related to parental care, especially for females with single‐parent care, have differences in encephalon size.

Among the studies evaluating sexual dimorphism and encephalic morphology for neotropical cichlids, there are only recent records for *Geophagus sveni* (de Oliveira & da Graça, 2020), of which the authors observed no statistical differences in encephalon morphology associated with sexual dimorphism.

Dimorphic characters associated with the morphology of areas linked to the nervous system of other Neotropical fish groups have been explored more recently. There is evidence of differences associated with sexual dimorphism in the olfactory system of Acestrorhamphidae (Characiformes): *Tyttobrycon shibattai* (Abrahão et al., 2019). The absence of differences associated with the olfactory system in cichlids is putatively associated with the group's conserved microsmatic condition (Striedter, 2005).

Regarding the non‐difference between encephalic subregions and the sexual dimorphism of Cichlinae, the accumulated knowledge on the subject is too early to assess whether factors associated with sexual selection or the type of parental care directly influence the encephalic morphology of neotropical lineages, in contrast to African lineages.

Studies involving neuroanatomical data and the relationship with the natural history of neotropical cichlids are still in the initial stages, representing a challenge insert to comparative biology. On the other hand, the Neotropical region has complex and heterogeneous habitats, such as rapid streams, which provide different selective pressures from the events that occurred in the great African lakes, since in these lakes, potentially the selective pressures are more linked to intense ecological interactions and niche occupation, rather than habitat diversity, which contrasts with the freshwater environments of the Neotropical region. Research on this subject is timely and promising, given the group's morphological, behavioral, and trophic diversity and the virtual occupation of cichlids in aquatic environments distributed throughout the various biomes of Central and South America.

## AUTHOR CONTRIBUTIONS

**Renan Leão‐Reis:** Conceptualization; investigation; methodology; data curation; project administration; writing – original draft; writing – review and editing; formal analysis. **Keila Xavier Magalhães:** Writing – review and editing; methodology; data curation; writing – original draft. **Thiago Nilton A. Pereira:** Writing – original draft; methodology; writing – review and editing; formal analysis. **Janice Muriel‐Cunha:** Writing – original draft; funding acquisition; writing – review and editing. **Alberto Akama:** Conceptualization; supervision; funding acquisition; writing – review and editing; writing – original draft.

## FUNDING INFORMATION

This project was supported by Conselho Nacional de Desenvolvimento Científico e Tecnológico (CNPq) for awarding a doctoral scholarship to Renan Leão‐Reis (#Proc. 140641/2020‐3). Alberto Akama was supported by Fundo de Defesa de Direitos Difusos program of the Brazilian Ministry of Justice and Public Safety (FDD 24/2019—0800.012635/2019‐80). Janice Muriel‐Cunha was supported by Amazonia +10 funded by Fundação Amazônia Paraense de Amparo à Pesquisa‐FAPESPA (proc. 103/2023) and INCT‐Peixes funded by Ministério da Ciência, Tecnologia e Inovação‐ MCTIC/CNPq (proc. 405706/2022‐7).

## CONFLICT OF INTEREST STATEMENT

The authors declare no conflict of interest.

## Supporting information


**TABLE S1.** Morphometric linear measurements of the sub‐regions used for respective volumetric conversion. F, female; HCCEB, height of *corpus cerebelli*; HDI, height of diencephalon; HTEO, height of *tectum mesencephali* + *torus semicircularis*; HTEL, height of telencephalon; HL, head length; IC, individual code; LCCEB, length of *corpus cerebelli*; LDI, length of diencephalon; LTEO, length of *tectum mesencephali + torus semicircularis*; LTEL, length of telencephalon; M, male; Me, mean; SL, standard length; TC, *Teleocichla centrarchus*; TG, *Teleocichla gephyrogramma*; THE, total encephalon height; TLE, total encephalon length; TM, *Teleocichla monogramma*; TWE, total encephalon width; WCCEB, width of *corpus cerebelli*; WDI, width of diencephalon; WTEO, width of *tectum mesencephali* + *torus semicircularis*; WTEL, width of telencephalon.

**TABLE S2.** Measurements of the specimens and the converted volumes of the encephalon and its sub‐regions. Values expressed are proportional to the total encephalon volume. BM, body mass; CCEB, volume of *corpus cerebelli* + *eminentia granularis*; DIE, volume of diencephalon; EM, encephalon mass; EV, encephalon volume; F, female; HL, head length; IC, individual code; M, male; SL, standard length; TC, *Teleocichla centrarchus*; TeO, volume of *tectum mesencephali* + *torus semicircularis*; TG, *Teleocichla gephyrogramma*; TLE, total encephalon length; TM, *Teleocichla monogramma*; TP*, *Teleocichla proselytus* data from de Oliveira and da Graça (2024); TEL, volume of telencephalon.
