# Supplementary material for: Neuroanatomical diversity in Teleocichla with new volumetric and histological insights into the encephalon of Teleocichla monogramma Kullander 1988
Source: Anat Rec (Hoboken). 2025 Nov 13;309(10):2897–912. doi: 10.1002/ar.70095 (PMC13549279; doi:10.1002/ar.70095)
Supplement: Supplementary file 1 [file AR-309-2897-s001.docx]

**TABLE 1S.** Morphometric linear measurements of the sub-regions used for respective volumetric conversion. Legends: F = female; HCCEB = height of *corpus cerebelli*; HDI = height of diencephalon; HTEO = height of *tectum mesencephali* + *torus semicircularis*; HTEL = height of telencephalon; HL = head length; IC = individual code; LCCEB = length of *corpus cerebelli*; LDI = length of diencephalon; LTEO = length of *tectum mesencephali* *+ torus semicircularis*; LTEL = length of telencephalon; M = male; Me = mean; SL = standard length; TC = *Teleocichla centrarchus*; TG = *Teleocichla gephyrogramma*; THE = total encephalon height; TLE = total encephalon length; TM = *Teleocichla monogramma*; TWE = total encephalon width; WCCEB = width of *corpus cerebelli*; WDI = width of diencephalon; WTEO = width of *tectum mesencephali* + *torus semicircularis*; WTEL = width of telencephalon.

| **IC** | **Sex** | **SL (mm)** | **HL (mm)** | **TLE (mm)** | **TWE (mm)** | **THE (mm)** | **L** **CCEB (mm)** | **W** **CCEB (mm)** | **H** **CCEB (mm)** | **LTEO (mm)** | **WTEO (mm)** | **HTEO (mm)** | **LDI (mm)** | **WDI (mm)** | **HDI (mm)** | **LTEL (mm)** | **WTEL (mm)** | **HTEL (mm)** |
| --- | --- | --- | --- | --- | --- | --- | --- | --- | --- | --- | --- | --- | --- | --- | --- | --- | --- | --- |
| TM3 | F | 39.7 | 9.3 | 2.86 | 2.2 | 1.49 | 0.9 | 0.76 | 0.73 | 1.47 | 1.14 | 1.01 | 1.01 | 1.45 | 0.42 | 1 | 0.8 | 0.82 |
| TM5 | F | 40.8 | 10 | 2.91 | 2.24 | 1.46 | 0.85 | 0.79 | 0.75 | 1.45 | 1.13 | 0.94 | 1.09 | 1.39 | 0.39 | 1.05 | 0.83 | 0.69 |
| TM6 | F | 47.7 | 12 | 3.32 | 2.38 | 1.62 | 1.04 | 1.1 | 0.82 | 1.59 | 1.18 | 1.08 | 1 | 1.49 | 0.41 | 1.15 | 0.88 | 0.78 |
| TM8 | F | 41.3 | 10.4 | 2.95 | 2.33 | 1.55 | 0.88 | 0.87 | 0.72 | 1.46 | 1.16 | 1.05 | 1.05 | 1.47 | 0.48 | 1.15 | 0.82 | 0.79 |
| TM10 | F | 34.3 | 9.7 | 2.52 | 2.05 | 1.31 | 0.82 | 0.76 | 0.63 | 1.4 | 1.06 | 0.9 | 0.96 | 1.27 | 0.35 | 0.93 | 0.7 | 0.69 |
| TM11 | F | 34.5 | 8.8 | 2.18 | 1.76 | 1.2 | 0.68 | 0.6 | 0.63 | 1.17 | 0.89 | 0.8 | 0.77 | 0.99 | 0.34 | 0.74 | 0.55 | 0.49 |
| TM13 | F | 36.4 | 9.4 | 2.30 | 1.79 | 1.12 | 0.65 | 0.62 | 0.57 | 1.19 | 0.92 | 0.79 | 0.76 | 1.03 | 0.28 | 0.81 | 0.58 | 0.53 |
| TM16 | F | 38.2 | 9.1 | 2.34 | 1.74 | 1.2 | 0.64 | 0.61 | 0.52 | 1.2 | 0.87 | 0.78 | 0.7 | 1.08 | 0.33 | 0.79 | 0.6 | 0.46 |
| TM19 | F | 35.8 | 9 | 2.23 | 1.73 | 1.17 | 0.65 | 0.69 | 0.58 | 1.22 | 0.88 | 0.78 | 0.76 | 1.06 | 0.36 | 0.77 | 0.59 | 0.51 |
| TM20 | F | 37.1 | 8.8 | 2.44 | 1.8 | 1.22 | 0.67 | 0.66 | 0.61 | 1.27 | 0.91 | 0.87 | 0.77 | 1.3 | 0.26 | 0.78 | 0.64 | 0.55 |
| TM21 | F | 44.8 | 10.8 | 2.62 | 2.14 | 1.37 | 0.88 | 0.74 | 0.64 | 1.32 | 0.96 | 0.84 | 0.86 | 1.29 | 0.41 | 0.83 | 0.63 | 0.6 |
| TM23 | F | 39.2 | 9.5 | 2.68 | 2.08 | 1.37 | 0.81 | 0.79 | 0.69 | 1.43 | 1 | 1.02 | 0.89 | 1.24 | 0.33 | 0.99 | 0.69 | 0.67 |
| TM24 | F | 43.1 | 10.2 | 2.62 | 2.13 | 1.31 | 0.82 | 0.73 | 0.73 | 1.36 | 1.05 | 0.91 | 0.86 | 1.31 | 0.32 | 0.91 | 0.72 | 0.65 |
| TM1 | M | 44.3 | 10.5 | 2.66 | 2.08 | 1.38 | 0.71 | 0.74 | 0.66 | 1.44 | 1 | 0.93 | 1.01 | 1.27 | 0.44 | 1.09 | 0.64 | 0.73 |
| TM2 | M | 40.5 | 10.2 | 2.83 | 2.12 | 1.49 | 0.87 | 0.82 | 0.68 | 1.37 | 1.07 | 1.15 | 0.93 | 1.35 | 0.46 | 1,07 | 0.74 | 0.63 |
| TM4 | M | 38 | 9.7 | 2.77 | 2.05 | 1.2 | 0.88 | 0.81 | 0.71 | 1.46 | 1.02 | 1.01 | 0.84 | 1.29 | 0.41 | 1.01 | 0.75 | 0.67 |
| TM7 | M | 46.8 | 11.7 | 3.06 | 2.31 | 1.59 | 0.95 | 0.96 | 0.8 | 1.55 | 1.23 | 1.08 | 0.95 | 1.47 | 0.39 | 1.1 | 0.79 | 0.82 |
| TM9 | M | 34.3 | 8.1 | 2.55 | 2 | 1.36 | 0.72 | 0.74 | 0.64 | 1.33 | 1.01 | 0.89 | 0.95 | 1.27 | 0.43 | 0.9 | 0.67 | 0.61 |
| TM12 | M | 48.4 | 11.8 | 2.65 | 2.11 | 1.49 | 0.84 | 0.75 | 0.73 | 1.47 | 1.16 | 0.9 | 0.94 | 1.34 | 0.42 | 0.92 | 0.71 | 0.68 |
| TM14 | M | 36.4 | 9.1 | 2.20 | 1.84 | 1.17 | 0.64 | 0.54 | 0.55 | 1.21 | 0.94 | 0.76 | 0.84 | 1.07 | 0.33 | 0.78 | 0.59 | 0.54 |
| TM15 | M | 38.3 | 9.9 | 2.38 | 1.91 | 1.2 | 0.7 | 0.66 | 0.61 | 1.34 | 0.98 | 0.81 | 0.89 | 1.21 | 0.37 | 0.78 | 0.6 | 0.54 |
| TM17 | M | 35.9 | 8.8 | 2.44 | 1.88 | 1.33 | 0.71 | 0.68 | 0.55 | 1.41 | 0.95 | 0.87 | 0.94 | 1.16 | 0.44 | 0.8 | 0.58 | 0.58 |
| TM22 | M | 39.9 | 11.3 | 2.70 | 2.03 | 1.33 | 0.76 | 0.8 | 0.67 | 1.42 | 1 | 0.94 | 0.87 | 1.29 | 0.41 | 0.98 | 0.68 | 0.69 |
| TG25 | - | 36.26 | 9.01 | 4.08 | 3.18 | 2.33 | 1.34 | 1.49 | 1.05 | 2.11 | 1.54 | 1.56 | 1.64 | 2.07 | 0.72 | 1.58 | 1.16 | 1.06 |
| TG27 | - | 29.32 | 7.54 | 3.55 | 2.9 | 2.01 | 1.11 | 1.19 | 1.1 | 1.92 | 1.46 | 1.32 | 1.45 | 1.73 | 0.6 | 1.54 | 1.04 | 0.99 |
| TG28 | - | 27.46 | 7.16 | 3.63 | 2.71 | 1.98 | 1.04 | 1 | 0.88 | 1.88 | 1.34 | 1.25 | 1.32 | 1.6 | 0.59 | 1.5 | 1.01 | 0.95 |
| TC30 | - | 36.56 | 9.15 | 4.14 | 3.21 | 2.24 | 1.28 | 1.15 | 0.88 | 2.16 | 1.67 | 1.56 | 1.5 | 2.03 | 0.62 | 1.53 | 1.07 | 1.1 |
| TC31 | - | 30.13 | 8.25 | 3.65 | 2.87 | 1.99 | 1.16 | 1.12 | 0.82 | 1.79 | 1.35 | 1.33 | 1.34 | 1.7 | 0.62 | 1.39 | 1.03 | 1.04 |
| TC32 | - | 31.43 | 8.57 | 3.76 | 3.19 | 2.06 | 1.21 | .122 | 0.83 | 1.99 | 1.54 | 1.48 | 1.35 | 1.96 | 0.66 | 1.52 | 1.08 | 1.08 |
