# Supplementary material for: Neuroanatomical diversity in Teleocichla with new volumetric and histological insights into the encephalon of Teleocichla monogramma Kullander 1988
Source: Anat Rec (Hoboken). 2025 Nov 13;309(10):2897–912. doi: 10.1002/ar.70095 (PMC13549279; doi:10.1002/ar.70095)
Supplement: Supplementary file 2 [file AR-309-2897-s002.docx]

**TABLE 2S.** Measurements of the specimens and the converted volumes of the encephalon and its sub-regions. Values expressed are proportional to the total encephalon volume. Legends: BM = body mass; CCEB = volume of *corpus cerebelli* + *eminentia granularis*; DIE = volume of diencephalon; EM = encephalon mass; EV = encephalon volume; F = female; HL = head length; IC = individual code; M = male; SL = standard length; TC = *Teleocichla centrarchus*; TeO = volume of *tectum mesencephali* + *torus semicircularis*; TG = *Teleocichla gephyrogramma*; TLE = total encephalon length; TM = *Teleocichla monogramma*; TP* = *Teleocichla proselytus* data from de Oliveira, da Graça (2024); TEL = volume of telencephalon.

| **IC** | **Sex** | **SL (mm)** | **HL (mm)** | **BM (g)** | **EM (g)** | **TLE (mm)** | **EV (mm^3^)** | **CCEB (mm^3^)** | **TEO (mm^3^)** | **DIE (mm^3^)** | **TEL (mm^3^)** | **CCEB (%)** | **TeO (%)** | **DIE (%)** | **TEL (%)** |
| --- | --- | --- | --- | --- | --- | --- | --- | --- | --- | --- | --- | --- | --- | --- | --- |
| TM3 | F | 39.7 | 9.3 | 0.65 | 0.0151 | 2.86 | 4.91 | 0.26 | 1.77 | 0.32 | 0.69 | 5.30% | 36.05% | 6.52% | 14.05% |
| TM5 | F | 40.8 | 10 | 0.71 | 0.0161 | 2.91 | 4.98 | 0.26 | 1.61 | 0.31 | 0.63 | 5.22% | 32.33% | 6.22% | 12.65% |
| TM6 | F | 47.7 | 12 | 1.14 | 0.0207 | 3.32 | 6.70 | 0.49 | 2.12 | 0.32 | 0.83 | 7.31% | 31.64% | 4.78% | 12.39% |
| TM8 | F | 41.3 | 10.4 | 0.78 | 0.0174 | 2.95 | 5.58 | 0.29 | 1.86 | 0.39 | 0.78 | 5.20% | 33.33% | 6.99% | 13.98% |
| TM10 | F | 34.3 | 9.7 | 0.45 | 0.0116 | 2.52 | 3.54 | 0.21 | 1.4 | 0.22 | 0.47 | 5.93% | 39.55% | 6.21% | 13.28% |
| TM11 | F | 34.5 | 8.8 | 0.38 | 0.0083 | 2.18 | 2.41 | 0.13 | 0.87 | 0.14 | 0.21 | 5.39% | 36.10% | 5.81% | 8.71% |
| TM13 | F | 36.4 | 9.4 | 0.45 | 0.0102 | 2.30 | 2.41 | 0.12 | 0.91 | 0.11 | 0.26 | 4.98% | 37.76% | 4.56% | 10.79% |
| TM16 | F | 38.2 | 9.1 | 0.56 | 0.0116 | 2.34 | 2.56 | 0.11 | 0.85 | 0.13 | 0.23 | 4.30% | 33.20% | 5.08% | 8.98% |
| TM19 | F | 35.8 | 9 | 0.45 | 0.0098 | 2.23 | 2.36 | 0.14 | 0.88 | 0.15 | 0.24 | 5.93% | 37.29% | 6.36% | 10.17% |
| TM20 | F | 37.1 | 8.8 | 0.47 | 0.011 | 2.44 | 2.80 | 0.14 | 1.05 | 0.14 | 0.29 | 5% | 37.50% | 5% | 10.36% |
| TM21 | F | 44.8 | 10.8 | 0.86 | 0.0145 | 2.62 | 4.02 | 0.22 | 1.11 | 0.24 | 0.33 | 5.47% | 27.61% | 5.97% | 8.21% |
| TM23 | F | 39.2 | 9.5 | 0.69 | 0.0137 | 2.68 | 3.99 | 0.23 | 1.53 | 0.19 | 0.48 | 5.76% | 38.35% | 4.76% | 12.03% |
| TM24 | F | 43.1 | 10.2 | 0.75 | 0.0134 | 2.62 | 3.83 | 0.23 | 1.36 | 0.19 | 0.45 | 6.01% | 35.51% | 4.96% | 11.75% |
| TM1 | M | 44.3 | 10.5 | 0.9 | 0.0149 | 2.66 | 3.99 | 0.18 | 1.4 | 0.3 | 0.53 | 4.52% | 35.18% | 7.54% | 13.32% |
| TM2 | M | 40.5 | 10.2 | 0.7 | 0.015 | 2.83 | 4.68 | 0.25 | 1.76 | 0.3 | 0.52 | 5.34% | 37.61% | 6..41% | 11.11% |
| TM4 | M | 38 | 9.7 | 0.63 | 0.0152 | 2.77 | 3.57 | 0.26 | 1.57 | 0.23 | 0.53 | 7.28% | 43.98% | 6.44% | 14.85% |
| TM7 | M | 46.8 | 11.7 | 0.99 | 0.0188 | 3.06 | 5.88 | 0.38 | 2.16 | 0.29 | 0.75 | 6.46% | 36.73% | 4.93% | 12.76% |
| TM9 | M | 34.3 | 8.1 | 0.53 | 0.013 | 2.55 | 3.63 | 0.18 | 1.25 | 0.27 | 0.38 | 4.96% | 34.44% | 7.44% | 10.47% |
| TM12 | M | 48.4 | 11.8 | 1.15 | 0.016 | 2.65 | 4.36 | 0.24 | 1.61 | 0.28 | 0.46 | 5.50% | 36.93% | 6.41% | 10.55% |
| TM14 | M | 36.4 | 9.1 | 0.56 | 0.0092 | 2.20 | 2.48 | 0.1 | 0.9 | 0.16 | 0.26 | 4.03% | 36.29% | 6.45% | 10.48% |
| TM15 | M | 38.3 | 9.9 | 0.56 | 0.01 | 2.38 | 2.85 | 0.15 | 1.11 | 0.21 | 0.26 | 5.26% | 38.95% | 7.37% | 9.12% |
| TM17 | M | 35.9 | 8.8 | 0.47 | 0.0102 | 2.44 | 3.19 | 0.14 | 1.22 | 0.25 | 0.28 | 4.39% | 38.24% | 7.84% | 8.78% |
| TM22 | M | 39.9 | 11.3 | 0.72 | 0.0155 | 2.70 | 3.81 | 0.21 | 1.4 | 0.24 | 0.48 | 5.51% | 36.75% | 6.30% | 12.60% |
| TG25 | - | 36.26 | 9.01 | 0.55 | 0.0097 | 4.08 | 15.82 | 1.10 | 5.31 | 1.28 | 2.03 | 6.93% | 33.54% | 8.09% | 12.85% |
| TG27 | - | 29.32 | 7.54 | 0.30 | 0.0070 | 3.55 | 10.83 | 0.76 | 3.87 | 0.79 | 1.66 | 7.02% | 35.76% | 7.27% | 15.32% |
| TG28 | - | 27.46 | 7.16 | 0.24 | 0.059 | 3.63 | 10.19 | 0.48 | 3.30 | 0.65 | 1.51 | 4.70% | 32.33% | 6.40% | 14.78% |
| TC30 | - | 36.56 | 9.15 | 0.50 | 0.0095 | 4.14 | 15.58 | 0.68 | 5.89 | 0.99 | 1.88 | 4.35% | 37.81% | 6.34% | 12.10% |
| TC31 | - | 30.13 | 8.25 | 0.25 | 0.0064 | 3.65 | 10.91 | 0.56 | 3.36 | 0.99 | 1.56 | 5.11% | 30.83% | 9.06% | 14.29% |
| TC32 | - | 31.43 | 8.57 | 0.30 | 0.0079 | 3.76 | 12.93 | 0.64 | 4.75 | 0.74 | 1.86 | 4.96% | 36.71% | 5.72% | 14.35% |
| TP* | - | 16.85 | - | - | - | - | 30.17 | 1.46 | 7.36 | 2.12 | 2.89 | 4.8% | 24.4% | 7% | 9.6% |
